# Host selection and potential predation in the host-parasite interaction between the isopod *Tachaea chinensis* and freshwater host species

**DOI:** 10.1016/j.ijppaw.2023.04.006

**Published:** 2023-04-15

**Authors:** Al-Wahaibi Mohamed Khalfan, Shotaro Tani, Yudai Aoki, Abdelgayed Younes, Hidetoshi Saito

**Affiliations:** a Department of Bioresource Science, Graduate School of Integrated Sciences for Life, Hiroshima University, 1-4-4 Kagamiyama, Higashihiroshima, Hiroshima, 739-8528, Japan; b Center of Excellence in Marine Biotechnology, Sultan Qaboos University, P.O Box 50, Al Khod 123, Muscat, Sultanate of Oman; c Hydrobiology Department, National Research Centre, Dokki 33 El-Buhouth Street, Egypt

**Keywords:** Host specificity, Parasite consumption, Pairwise experiments, *Palaemon paucidens*, *Macrobrachium nipponense*, *Procambarus clarkii*

## Abstract

*Tachaea chinensis* is an ectoparasite commonly found on diverse ecologically and commercially valuable freshwater shrimps and prawns. Previous studies on this parasite have focused on its distribution and taxonomical identification, while its host preference and/or the potential predation in this host-parasite interaction remained poorly understood. In this study, we investigate the host preference and potential predation of the isopod *T. chinensis* using manipulative choice and predation experiments under laboratory settings. The preference toward a broad range of host decapods in single-host treatments, indicates a low host specificity, which ultimately aids in the survival of this parasite in the natural environment. *Tachaea chinensis* responded well to the shrimp *Palaemon paucidens* when presented with uncommon host species in all three treatments. In host-parasite predation treatments, all the tested *P. paucidens* shrimp, the prawn *Macrobrachium nipponense*, and the crayfish *Procambarus clarkii* were able to consume the isopod–especially the invasive crayfish *P. clarkii*, which consumed a greater percentage in a considerably shorter time frame (Fisher's exact test, *P* < 0.01). This study demonstrated for the first time the ability of larger freshwater decapods to prey upon *T. chinensis*. Despite the large difference in the maximum attainable size of those freshwater species, a high predation pressure by the invasive crayfish on the isopod is anticipated, if they are present in the same environment.

## Highlights

• The isopod *Tachaea chinensis* infested all of the tested decapods species.
• The artificial shrimp and fish species received the lowest affinity by the isopod.
• *Tachaea chinensis* host selection strongly favor the common host *Palaemon paucidens*.
• Large host species predated on *Tachaea chinensis* isopod in laboratory settings.
• The invasive crayfish *Procambarus clarkii* consumed the isopod in shorter time.

## Graphical abstract

**Figure:**
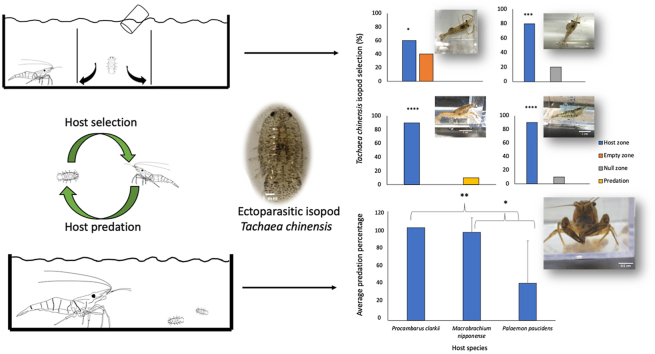

## 1 Introduction

*Tachaea chinensis* Thielemann, 1910 is an ectoparasitic isopod infesting freshwater shrimps and prawns in Japan, China, Vietnam, Thailand, and Malaysia (Shiino, 1965; Nagasawa et al., 2018; Xu et al., 2019, 2021; Nunomura and Shimomura, 2020). It exhibits a biphasic lifestyle– isopods in the manca stage and immature stage infest the host shrimps, whereas during the mature stages, they detach for reproductive proposes (Ota, 2019). The manca stage passes through five distinct embryonic stages within 30–38 days, although the period from the manca stage to the mature adult stage may span 180 days on average (Xu et al., 2021). *Tachaea chinensis* is commonly found adhering to the carapace of shrimps, which can cause various physiological effects and potential secondary infections (Nagasawa et al., 2018; Li et al., 2019a, 2019b, 2020; Ueki et al., 1988).

Parasites-host specialization differs by geographical locations, whereby host density may play an important role in the observed infestation pattern (Ota, 2019). In Japan, the isopod *T. chinensis* is found on various host species depending on the density of the host species at a given site, but they are frequently observed infesting the shrimp *Palaemon paucidens* De Hann, 1844, in the Honshu region in Japan's mainland (Nagasawa et al., 2018). This isopod has also been found infesting the exotic shrimp *Palaemon sinensis* (Sollaud, 1911) in Japan (Oonuki et al., 2010; Imai and Oonuki, 2014; Saito, 2017, 2018; Saito et al., 2016, 2019; Imai et al., 2021; Ogasawara et al., 2021), as well as *P. sinensis* in rice fields in China (Xu et al., 2019). Other potential hosts include the prawn *Macrobrachium nipponense* (De Haan, 1849), the invasive crayfish *Procambarus clarkii* (Girard, 1852), and freshwater fishes occupying similar habitats. While the association between these prawn and crayfish is rare in nature, the infestation on fish species is considered accidental (Shen, 1936; Nagasawa et al., 2018). According to previous behavioral observations, the isopod immediately approached the prawn *M*. *nipponense* after being released into a tank, which indicates that it has strong visual capabilities (Nagasawa and Fujimoto, 2021). Similar observations were made in another study, which concluded that the isopod might be incorporating different sensory mechanisms to locate its host; however, the sensory attractant was difficult to pinpoint in both studies owing to the presence of different sensory stimuli in the experimental tank (e.g., visual, chemical, and tactile) (Khalfan et al., 2022).

Parasites–host interactions are usually governed by environmental and biological factors (Nagel and Grutter, 2007). However, host size and overall surface area could resemble determinant factors for a successful association. In one study, individuals of *T. chinensis* were always found on host shrimps that are slightly larger in carapace length compared to its own body length (Khalfan et al., 2022). Similar findings were observed by Ota (2019), who concluded that the small-sized shrimp, *Neocaridina* spp., may not offer adequate space for the isopod to safely attach itself. Khalfan et al. (2023), using behavioral experiments, further elucidated this hypothesis and concluded that 0.7–1.0 ratios of parasite body length to host shrimp carapace length appeared to be preferred by the isopod *T. chinensis*, because it provides adequate space for safe attachment while avoiding potential consumption by the available host shrimps. In the same study, the authors found that relatively large size differences between the parasite body length and host shrimp carapace length resulted in the disappearance of the isopod from the experimental tank, potentially via predation by the large host shrimp. Incidences of host predation on the parasite were also observed in another study on *T. chinensis* isopods under laboratory conditions (Xu et al., 2021). Similar predation incidence was observed in gnathiid isopods. Different size classes of coral reef fishes were able to prey on the isopods, and larger fishes in particular consumed more isopods compared with their smaller counterparts (Penfold et al., 2008). Therefore, a higher probability of predation may exist with larger hosts, which may alter the isopods’ host choice. Nonetheless, whether these isopods are exhibiting host selection and/or predation mitigation patterns remains unclear.

In this study, we investigated the host-selection behavior by and host predation on *T. chinensis* isopods across different potential host species through pairwise choice experiments. A series of common versus uncommon host and predation experiments were conducted, under laboratory conditions, to help clarify these aspects in the host–parasite relationship of *T. chinensis*.

## 2 Materials and methods

### 2.1 Sampling of parasitic isopods and host species

The parasitic isopod *Tachaea chinensis* (Isopoda: Corallanidae); the freshwater host species *P. paucidens*, *P. sinensis*, *Neocaridina* spp., *M. nipponense*, *P. clarkii;* the Japanese rice fish *Oryzias latipes* (Temminck and Schlegel, 1846); and the introduced bitterling *Rhodeus ocellatus* (Kner, 1866) were collected from ponds and waterways in Okayama Prefecture and Shimane Prefecture, Japan, from August 2021 to January 2023. The parasites and host species were scooped up using hand nets (35 × 35 cm; 2.5-mm mesh size; 240-cm handle length) from the waterways’ side walls and submerged vegetation. Parasitized host shrimps and individual parasites were placed in water-filled containers fitted with aeration and transported to the laboratory of Aquatic Ecology at Hiroshima University.

In the laboratory, host shrimps infested with *T. chinensis* were acclimated in different acrylic tanks (58.5 cm × 15.5 cm × 21.5 cm, 57.5 cm × 26.8 cm × 30 cm, and 43 cm × 5 cm × 21 cm) according to host species. The water temperature in the acclimation tanks was maintained at 24 ± 1 °C and were on a 12D:12L automated light cycle. Host shrimps were fed daily with a commercial feed (Hikari Ranchu discs, 1.3–1.5 mm, KYORIN– Himeji, Hyogo, Japan). All treatments were conducted only on active shrimps and isopods within 1 week from sampling.

### 2.2 Host-species selection experiments

**Fig. 1:**
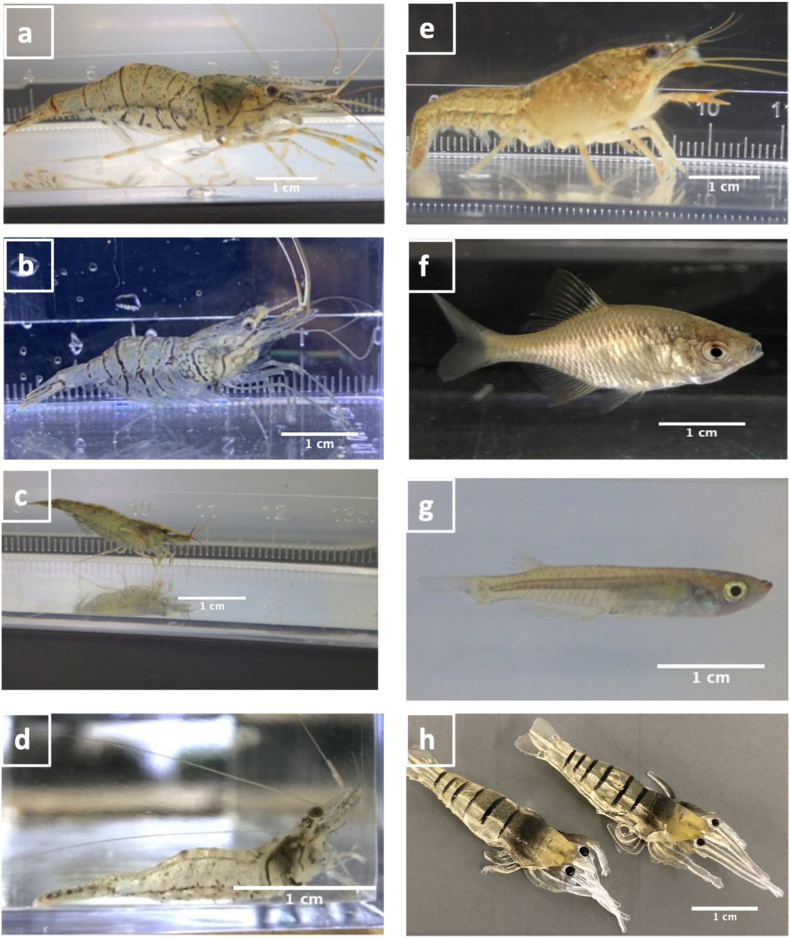
Eight different host options were used to investigate the host selection of *Tachaea. chinensis* isopods. (a) *Palaemon paucidens*; (b) *Palaemon sinensis*; (c) *Neocaridina* spp.; (d) *Macrobrachium nipponense*; (e) *Procambarus clarkii*; (f) *Rhodeus ocellatus*; (g) *Oryzias latipes* and (h) Artificial *P. paucidens*.

The experiments included a series of single-host and common versus uncommon host treatments. A total of eight treatments (Treatments 1–8) were included in the single-host experiment using different hosts, namely: *P. paucidens*, *P. sinensis*, *Neocaridina* spp., *Macrobrachium nipponense*, *P. clarkii*, an artificial *P. paucidens* (e.g., a soft plastic fishing lure imitating *P. paucidens,* 10-mm carapace length), *O. latipes*, and *R. ocellatus* (Table 1 and Fig. 1). For each host, ten trials were completed. These host species were selected based on their co-existence with *T. chinensis* at the sampling sites. Different isopod specimens were used for each treatment. The single-host treatments were conducted to investigate the isopods’ selection in the presence of only one host option in the tank. All isopods were retrieved from the common source host *P. paucidens* to avoid choice behavior being influenced by differences in source host.

**Table 1:** Experimental treatments to investigate the host preference behavior of *Tachaea chinensis*. Each treatment involved 10 replicates.

Treatments	Experiment category	Mean ratios between *Tachaea chinensis* body length and hosts carapace length	Host		
			Zone A^a^	Zone B	Number of trials
1	Single-host	0.8	*Palaemon paucidens*	Empty	10
2		0.9	*Palaemon sinensis*	Empty	10
3		1.0	*Neocaridina* spp.	Empty	10
4		0.9	*Macrobrachium nipponense*	Empty	10
5		0.8	*Procambarus clarkii*	Empty	10
6		0.7	Artificial *Palaemon paucidens*	Empty	10
7		-b	*Oryzias latipes*	Empty	10
8		–	*Rhodeus ocellatus*	Empty	10
9	Common versus un-common host	1.0	*Palaemon paucidens*	*Macrobrachium nipponense*	10
10		0.9	*Palaemon paucidens*	*Procambarus clarkii*	10
11		0.7	*Palaemon paucidens*	Artificial *Palaemon paucidens*	10

a Zones A and B indicates the two opposite ends of the experimental tank.

b *T. chinensis* average body length to fish total length ratio in both fish treatments was 0.2.

In the common versus uncommon host treatments, the uncommon hosts were the prawn *M. nipponense*, the invasive crayfish *P. clarkii,* and an artificial *P. paucidens* (fishing lures, HENGJIA Network Technology Co., Ltd, Jiading District, Shanghai, China) (Treatments 9–11). *Macrobrachium nipponense* and *P. clarkii* were selected based on their infrequency of infestation according to the literature and the list of preferred hosts for *T. chinensis* provided by Nagasawa et al. (2018). These trials were designed to compare the choice behavior of *T. chinensis* in the presence of an uncommon host that occurs in similar environments.

In both the single-host and common versus uncommon host treatments, shrimp carapace lengths and *T. chinensis* body lengths were measured to the nearest 0.1 cm using a digital caliper*.* Host species carapace length was measured from the orbital edge to the posterior margin. Meanwhile, isopod body length was measured from the border of the head to the end of the uropods. *Tachaea chinensis* ranged from 4 mm to 10 mm in body length. The ratio of the isopod body length to host carapace length was set between 0.7 and 1.0, as such a ratio is optimal to promote size selection in this parasite (Khalfan et al., 2023). The isopods were starved for 24 h prior to experimentation. All provided choice pairs had similar carapace lengths (e.g., those of shrimps) to avoid selection results being influenced by differences in host species sizes (Khalfan et al., 2023).

**Fig. 2:**
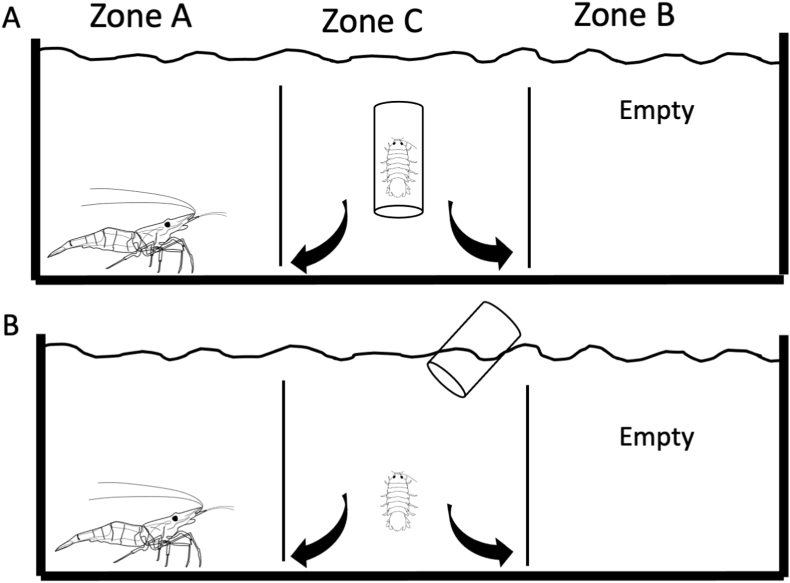
Schematic representation of the experimental system used to test the host selection behavior of the isopods in the single-host treatments. A: *Tachaea chinensis* at 20-min acclimation. B: *T. chinensis* after release.

**Fig. 3:**
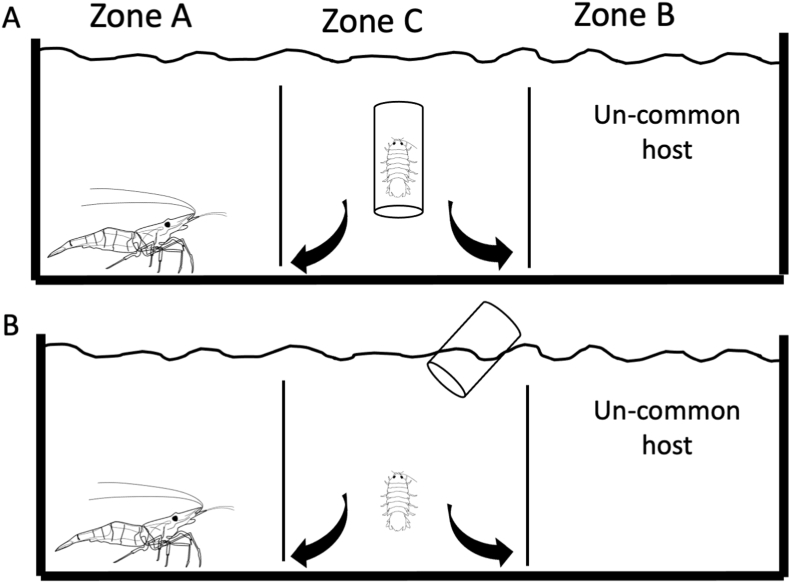
Schematic representation of the experimental system used to test the host selection behavior of the isopods in the common vs un-common host treatments. A: *Tachaea chinensis* at 20-min acclimation. B: *T. chinensis* after release.

The single-host and common-versus uncommon-host treatments were set up using small plexiglass tanks (17.5 cm × 8.4 cm × 6.5 cm) divided by two plastic barriers that allowed free passage of the isopods but prevented the passage of the experimental shrimps (Fig. 2, Fig. 3). In each treatment, the experimental tank was washed and filled with priorly dechlorinated tap water, to prevent the presence of chemical cues that might alter the choice selection behavior of the parasite. The host species (Table 1) were then placed alternatively at the opposite ends of the experimental tank (8.6 cm apart). After that, *T. chinensis* was introduced into the middle of the tank using a transparent glass container and allowed to acclimate for 20 min before release. To avoid possible escape behavior that could be mistaken for an actual preference, the experiment was run for 18 h after overnight exposure in both the single-host and common-versus uncommon-host treatments. The host preference was recorded based on the zone in which the isopods were found at each time point. If *T. chinensis* was found in Zone C after 18 h, the results were considered null. The parasite was considered to have chosen a host if it was found in Zone A or Zone B. Control trials (n = 10), without any choice object (e.g., empty tank), were conducted to determine whether the isopod had any preference for a certain direction/zone in the experimental tank. The results indicated no specific preference for either Zone A or Zone B.

### 2.3 Preliminary predation experiment

Additionally, preliminary experiments to evaluate the possibility of host predation on the isopods *T. chinensis* were conducted using the freshwater species *P. paucidens, M. nipponense,* and *P. clarkii* (Table 2). On the basis of its known destructive ability to freshwater vegetations and invertebrates in nature, we hypothesized that *P. clarkii* could impose greater predation pressure on *T. chinensis*.

**Table 2:** Experimental treatments to investigate the host predation on *Tachaea chinensis*. Each treatment involves 10 replicates.

Treatments	Mean ratios between *Tachaea chinensis* body length and hosts carapace length	Shrimp species	Number of trials
1	0.4	*Palaemon paucidens*	10
2	0.3	*Macrobrachium nipponense*	10
3	0.4	*Procambarus clarkii*	10

**Fig. 4:**
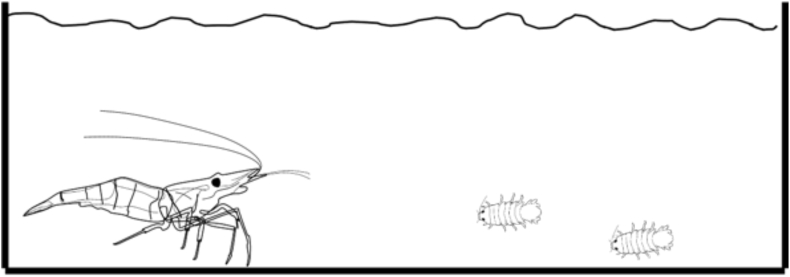
The experimental system used to test the potential predation of *Tachaea chinensis* by freshwater host species.

Thus, in each treatment, a single large host was provided with two *T. chinensis* of similar sizes in the same tank. Predation experiments were conducted using similar plexiglass tanks (17.5 cm × 8.4 cm x 6.5 cm) without any barriers to allow both the host species and isopod to move freely (Fig. 4). In each treatment, the ratios of mean body size to carapace length were set between 0.2 and 0.5 to investigate predation behavior by the host species. The number of *T. chinensis* consumed by each freshwater species was then recorded at each designated time point (1, 2, 3, 6, 12, 24 h). (See Fig. 3)

### 2.4 Statistical analysis

The binomial test of significance was used to compare the final position of the isopod against the theoretical probability of 50%, whereby the recorded results (e.g., host option A, host option B, no selection (null), and/or predation) had equal probabilities of occurrence. Fisher's exact test was used to investigate the relationship between the host species and the quantity of *T. chinensis* consumed in the predation experiments. All analyses were performed using R statistical software (Version 4.0.3, R foundation for statistical computing, Vienna, Austria).

## 3 Results

### 3.1 Single-host treatments

**Fig. 5:**
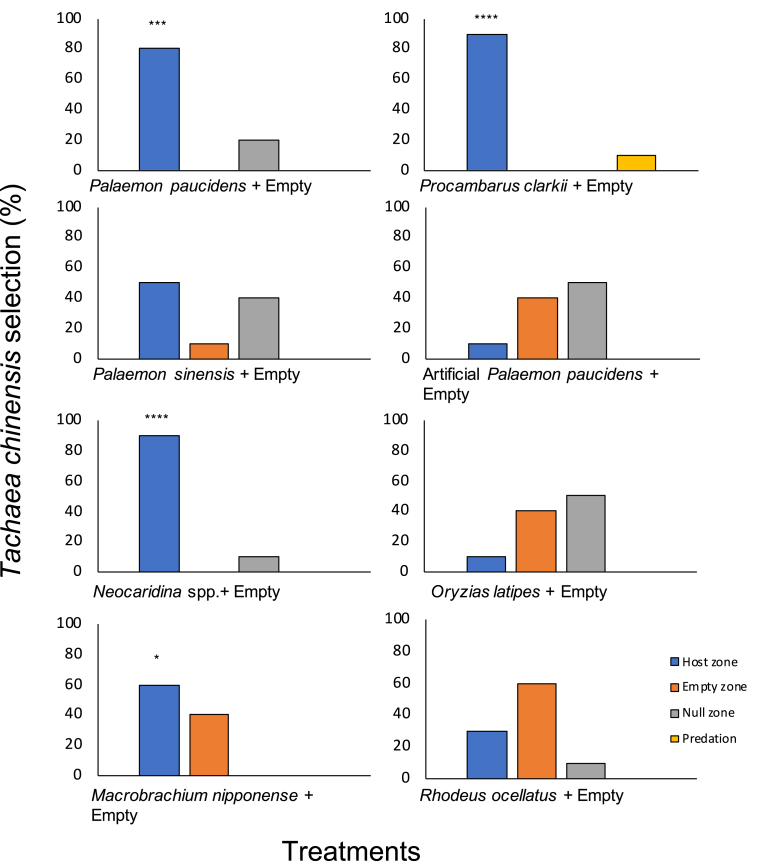
Selection percentage of *Tachaea chinensis* in the single-host treatments. Each treatment was repeated 10 times (one isopod per treatment); *: *P* < 0.05, ***: *P* < 0.001, ****: *P* < 0.0001 (Binomial test of significance).

The isopod *T. chinensis* showed significant selection behavior when provided with a single-host choice of *P. paucidens* (80% selection, 100% attachment; *P* < 0.001; binomial test of significance), *Neocaridina* spp. (90% selection, 100% attachment; *P* < 0.0001; binomial test of significance), *M. nipponense* (60% selection, 10% attachment; *P* < 0.05; binomial test of significance), and *P. clarkii* (90% selection, 100% attachment; *P* < 0.0001; binomial test of significance). However, it did not exhibit significant selection behavior when provided with *P. sinensis, O. latipes,* and *R. ocellatus* (Fig. 5). In the artificial *P. paucidens* treatment, although the isopod was found attracted to the host option soon after release, no significant selection behavior was recorded after 18 h.

### 3.2 Common versus uncommon hosts

**Fig. 6:**
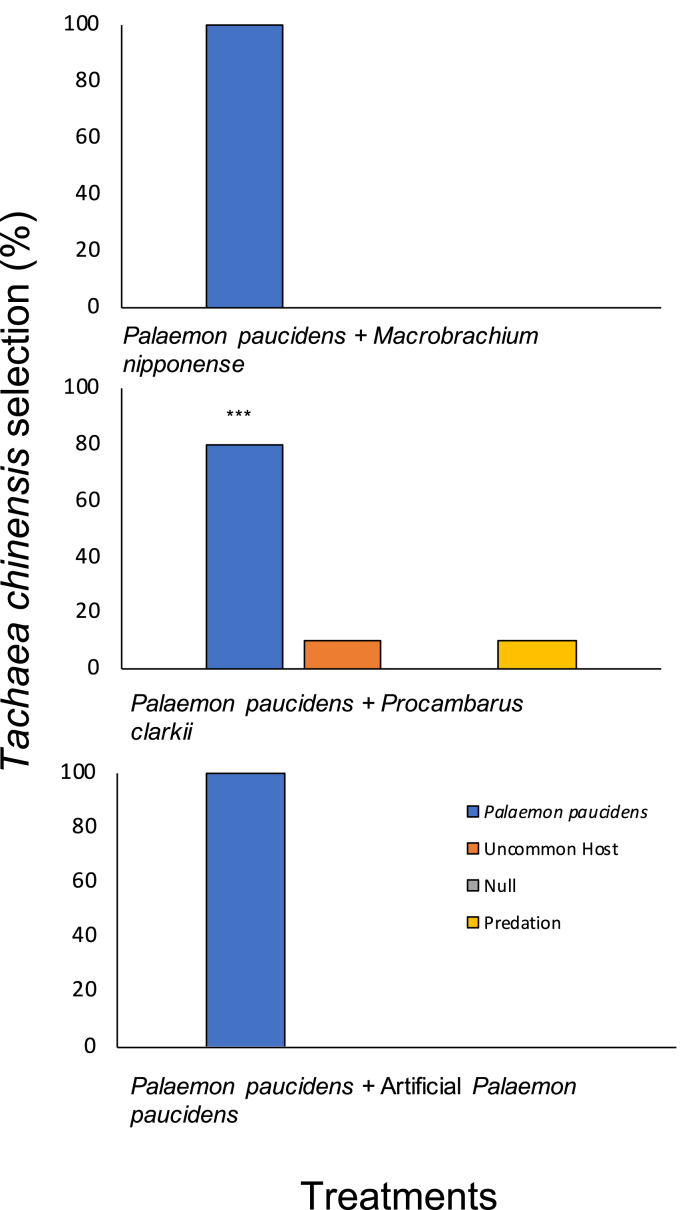
Selection percentage of *Tachaea chinensis* when subjected to un-common host selection experiments. Each treatment was repeated 10 times (one isopod per treatment); ***: *P* < 0.001 (Binomial test of significance).

Almost all the isopods were found to be significantly associated with the common host shrimps *P. paucidens* rather than *M. nipponense, P. clarkii*, and the artificial *P. paucidens* (Fig. 6), whereby the selection proportion scored 80% for the host-selection treatments involving *P. clarkii* (*P* < 0.001; binomial test of significance).

### 3.3 Preliminary predation experiment

**Fig. 7:**
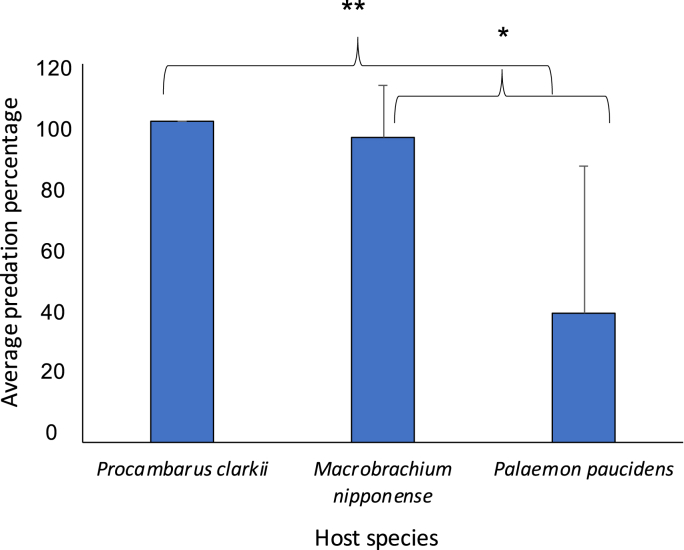
Average predation proportion of *Tachaea chinensis* in each freshwater decapod's species treatment. Fishers exact test, *P < 0.05, **P < 0.01.

**Fig. 8:**
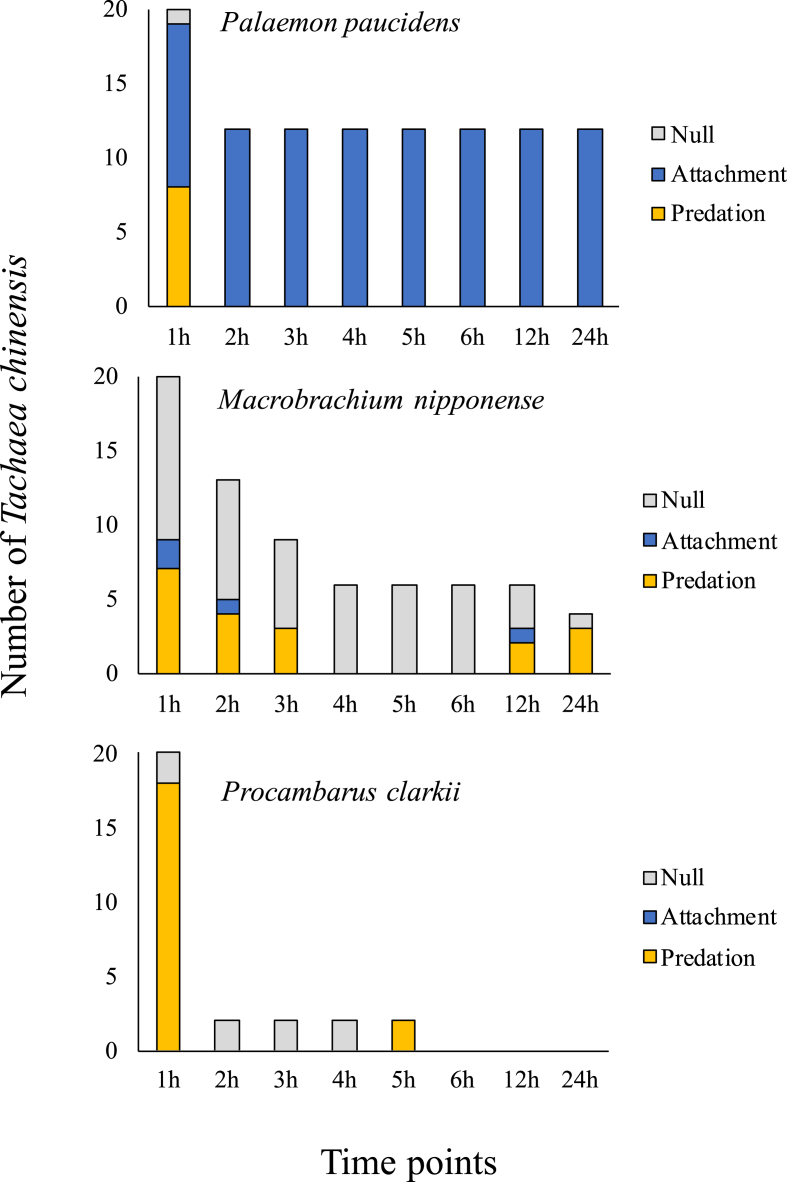
Number of *Tachaea chinensis* predated by; *Palaemon paucidens, Macrobrachium nipponense* and *Procambarus clarkii*. A total of 20 *T. chinensis* isopods (two isopods per trials, 10 replications) were used in each treatment.

In all three treatments, the host preyed on *T. chinensis* (Fig. 7). In treatments involving *P. clarkii*, 100% of the provided isopods were consumed after 24 h, mostly within 1 h of their introduction into the tank. Similarly, *M. nipponense* consumed 95% of the isopods 24 h after introduction. Meanwhile, *P. paucidens* had preyed on only 40% of the isopods after 24 h. The “null” results (e.g., parasites not attached to nor consumed by the host species) in the *P. paucidens* treatment were only observed for 1 h after release. On the contrary, the null results lingered for 24 h without being consumed nor showing attachment to the host species in the *M. nipponense* treatment. In *P. clarkii* treatment, the null results were observed for 4 h after release, after which all the isopods were consumed. There was a significant relationship between the number of isopods consumed and the host species for *P. clarkii* versus *P. paucidens* (Fisher's exact test, *P* < 0.01) and for *M. nipponense* versus *P. paucidens* (Fisher's exact test, *P* < 0.01)*.* Successful attachment between the isopod and the host species were found to be much higher with the common host shrimp *P. paucidens* (60%) than with *M. nipponense* (20%) or *P. clarkii* (0%) (Fig. 8).

## 4 Discussion

### 4.1 Host-selection treatments

**Fig. 9:**
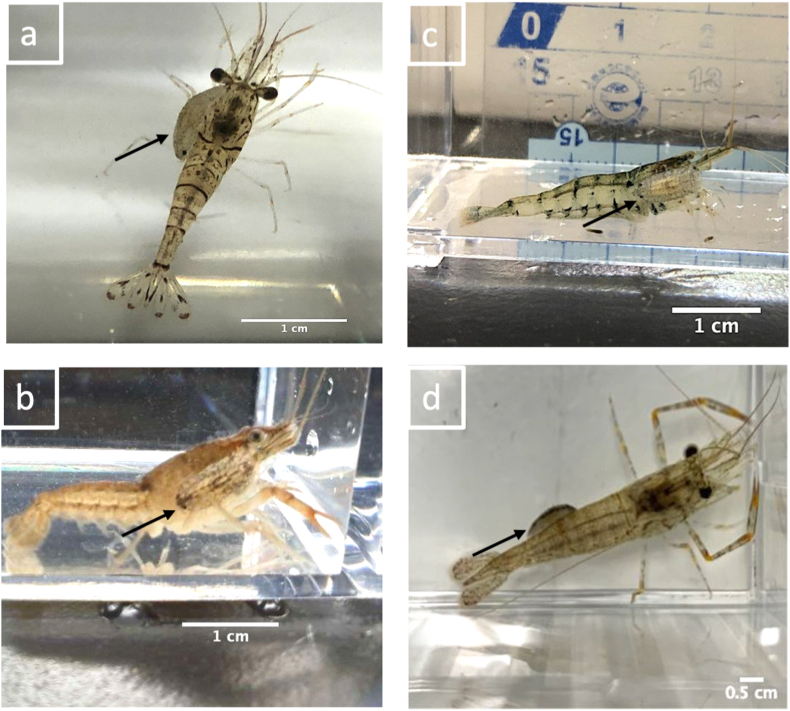
Attachments of *Tachaea chinensis* on various freshwater decapods during this study. The arrows indicate the position of the isopod on the host. (a) *T. chinensis* on the left-side of the carapace of *Palaemon paucidens*; (b) *T. chinensis* on the right-side of the carapace of *Procambarus clarkii*; (c) *T. chinensis* attached on the right-side of the carapace of *Neocaridina* spp.; and (d) *T. chinensis* initially clinging on the abdomen of *Macrobrachium nipponense*.

The ectoparasite *Tachaea chinensis* has been found infesting a broad range of host shrimps and prawns, including the three families Atyidae, Palaemonidae, and Penaeidae (Xu et al., 2019; Nagasawa et al., 2018). The observed host–parasite associations may not necessarily correspond to low host specificity of the isopods because such interactions are greatly influenced by biotic and abiotic factors (Nagel and Grutter, 2007). This is the first study to investigate the host preference of *T. chinensis* across different host species under manipulated laboratory conditions. Our findings reveal a strong preference by *T. chinensis* for the common host shrimp *P. paucidens* when uncommon host species (*M. nipponense, P. clarkii*, and artificial *P. paucidens*) are also present. They were found to show a significant selection preference for the source host *P. paucidens.* Surprisingly, however, in the control treatments (e.g., single-host treatments), *T. chinensis* were found to select the uncommon hosts at high frequencies. This indicates that even if only one host is available, as long as it is the proper size (e.g., medium size), *T. chinensis* could swiftly utilize the existing host shrimps to ensure its survival. The association of *T. chinensis* with *M. nipponense* and *P. clarkii* is rare in nature (Nagasawa et al., 2018). However, we clearly demonstrated the ability of this isopod to attach to this host prawn and invasive crayfish if it is presented with host sizes that do not jeopardize its survival (Fig. 9). Hosts that are small in size likely do not provide adequate space for an attachment whereas large host sizes could mean higher predation and thus mortality of the isopods (Khalfan et al., 2023).

The ability of *T. chinensis* to locate and distinguish appropriate host sizes could involve the use of a sophisticated visual system (Khalfan et al., 2023). Although no successful attachments were observed on the artificial shrimp, the isopod was found initially roaming around the it before eventually moving in the opposite direction. This indicates that the parasite has a strong visual ability that allows it to detect host objects in the experimental tank. The low host preference of *T. chinensis* for freshwater fish is unsurprising, given that any infestation of this isopod on fish in nature is usually considered accidental (Nagasawa et al., 2018).

The reason behind the significant preference of *T. chinensis* for *P. paucidens* may be related to the effects of the source host; that is, all the experimental isopods were retrieved from *P. paucidens*. Alternatively, the role of low predation potential cannot be ruled out. This host shrimp consumed the smallest number of isopods in this study, which suggests that it is a less harmful host species compared with the others. Therefore, the isopods’ host preference may be established from previous associations, and host preference of the subsequent stages may be determined by other biotic factors (e.g., food quality and low predation probability) (Nagel and Grutter, 2007). However, such a hypothesis is yet to be explored, as manca stage *T. chinensis,* upon hatching, have been found attaching to different species of host shrimp (Xu et al., 2021; Khalfan et al., 2023).

Isopod prevalence may be partially governed by the locality and type of species found at a given location (Nagasawa et al., 2018; Ota, 2019; Xu et al., 2019), which might explain the great variability in the selected host species in the single-host treatments. Therefore, *T. chinensis* isopod may demonstrate a high degree of flexibility with regard to host species and readily adapt to different hosts that provide adequate space for attachment. Previous studies from China and Japan have also come to similar conclusions (Ota, 2019; Xu et al., 2019).

### 4.2 Predation treatments

Apart from a single observational record of shrimps preying on the manca stage of *T. chinensis* in China (Xu et al., 2021), little information on the host–parasite relationship is available in the literature, particularly on the topic of host predation on *T. chinensis* isopods. This is the first record documenting the predation on this parasite by shrimp, prawn, and crayfish species under manipulated laboratory conditions. Laboratory trials on potential predation indicate that large *P. paucidens*, *M. nipponense*, and *P. clarkii* are able to consume *T. chinensis* isopods. Reports of predation on the parasite by the host were also documented in other isopod species including the gnathiid isopods (Penfold et al., 2008). In this experiment, *P. clarkii* crayfish and *M. nipponense* consumed more isopods compared with *P. paucidens*. The invasive crayfish also consumed the isopods rapidly, eliminating most of them within just 1 h.

**Fig. 10:**
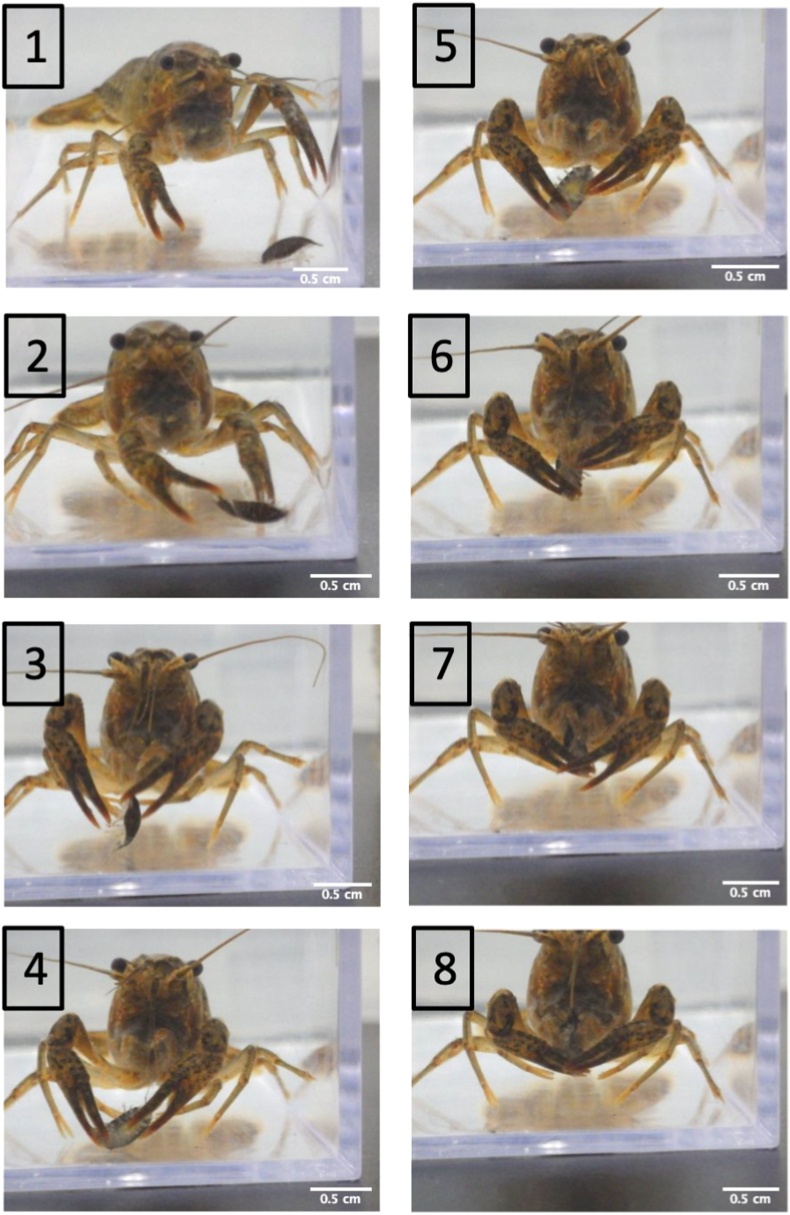
Prey handling procedure of the crayfish *Procambarus clarkii* (carapace length: 19 mm). (1) the crayfish *P. clarkii* approaching an 8 mm body length *Tachaea chinensis*; (2)–(5) *P. clarkii* catching and manipulating the prey using its pair of chelipeds; (6)–(8) the crayfish began consuming the prey by placing it directly into its mandibles.

The crayfish *P. clarkii* is a freshwater decapod native to North America. It was introduced as food for the American bullfrog *Rana catesbeiana* (Show, 1802) in Japan in 1927. Since then, it has shown great adaptability and has expanded its distribution across Japan (Kawai and Kobayashi, 2005). This crayfish consumes aquatic plants, fish eggs, aquatic insects, and macroinvertebrates, which ultimately leads to habitat degradation (Souty-Grosset et al., 2016; Watanabe and Ohba, 2022). Additionally, they were found to prey on animals of specific sizes, depending on their size (Machida and Akiyama, 2013). While the minimum size difference that triggers predation between the crayfish and the isopod has not been identified, our results suggest that predation only occurs when the size difference between the two organisms is large. We suspect that other potential factors influencing the isopod's susceptibility to predation include the strength and/or presence/absence of a large cheliped (Fig. 10). However, further studies are needed to help clarify the basis behind predation intensity of *P. clarkii* and other host species.

## 5 Conclusions

The current study demonstrated that, in the presence of uncommon hosts, the isopods showed a strong preference toward the source host species *P. paucidens*. However, *T. chinensis* was found infesting different hosts at varying degrees. The low host specificity found in this study complies with that of other isopod species in the family Corallanidae (Ho and Tonguthai, 1992; Gentil-Vasconcelos and Tavares-Dias, 2015) and other studies on *T. chinensis* (Xu et al., 2021; Ota, 2019; Nagasawa et al., 2018). This low host specificity likely facilitates the successful existence of this isopod in different habitats.

Moreover, potential predation by the host on the parasite was clearly demonstrated by large freshwater decapods including *M. nipponense, P. paucidens*, and *P. clarkii*. Although most of the tested shrimp preyed on the isopod, the invasive crayfish *P. clarkii* consumed a greater percentage over a shorter time. This suggests that there is an additional, often overlooked, destructive pressure by this invasive shrimp on freshwater ectoparasites.

## Author contributions

AMK contributed in the study design and execution. AMK, HS, ST and YA helped in sample collection. AMK and HS wrote the manuscript. HS, AY and ST edited and provided useful insights on experimental design and data analysis. AMK, HS, ST, YA and AY reviewed the final draft of this work and provided a genuine approval for publication.

## Declaration of competing interest

The authors declare that they have no conflict of interests.
